# Thrombospondin-1 null mice are resistant to hypoxia-induced pulmonary hypertension

**DOI:** 10.1186/1749-8090-5-32

**Published:** 2010-05-04

**Authors:** Cristhiaan D Ochoa, Lunyin Yu, Essam Al-Ansari, Charles A Hales, Deborah A Quinn

**Affiliations:** 1 Pulmonary and Critical Care Unit, Department of Medicine, Massachusetts General Hospital and Harvard Medical School, Boston, MA, USA

## Abstract

**Background and objective:**

Chronic hypoxia induces pulmonary hypertension in mice. Smooth muscle cell hyperplasia and medial thickening characterize the vasculature of these animals. Thrombospondin-1 null (TSP-1^-/-^) mice spontaneously develop pulmonary smooth muscle cell hyperplasia and medial thickening. In addition, TSP-1 produced by the pulmonary endothelium inhibits pulmonary artery smooth muscle cell growth. Based on these observations we sought to describe the pulmonary vascular changes in TSP-1^-/- ^mice exposed to chronic hypoxia.

**Methods:**

We exposed TSP-1^-/- ^and wild type (WT) mice to a fraction of inspired oxygen (FiO2) of 0.1 for up to six weeks. Pulmonary vascular remodeling was evaluated using tissue morphometrics. Additionally, right ventricle systolic pressures (RVSP) and right ventricular hypertrophy by right ventricle/left ventricle + septum ratios (RV/LV+S) were measured to evaluate pulmonary hypertensive changes. Finally, acute pulmonary vasoconstriction response in both TSP-1^-/- ^and WT mice was evaluated by acute hypoxia and U-46619 (a prostaglandin F2 analog) response.

**Results:**

In hypoxia, TSP-1^-/- ^mice had significantly lower RVSP, RV/LV+S ratios and less pulmonary vascular remodeling when compared to WT mice. TSP-1^-/- ^mice also had significantly lower RVSP in response to acute pulmonary vasoconstriction challenges than their WT counterparts.

**Conclusion:**

TSP-1^-/- ^mice had diminished pulmonary vasoconstriction response and were less responsive to hypoxia-induced pulmonary hypertension than their wild type counterparts. This observation suggests that TSP-1 could play an active role in the pathogenesis of pulmonary hypertension associated with hypoxia.

## Background

Thrombospondin-1 (TSP-1) is expressed at high levels in the heart, lungs, and liver of adult mice [1]. TSP-1 has been reported to both stimulate and inhibit endothelial cell and smooth muscle cell growth [2] smooth muscle migration [3,4], and to both stimulate and inhibit angiogenesis [5]. These contradictory findings are explained by the ability of TSP-1 to interact with various matrix proteins [6,7]. Consequently, TSP-1 has been implicated in the pathogenesis and treatment of a variety of human diseases including cancer [8], myocardial infarction [9] and peripheral vascular disease [10].

In the lung, clinical evidence suggests that TSP-1 could play a role in the pathogenesis of acute respiratory distress syndrome [11], idiopathic interstitial pneumonia [12] and chronic obstructive pulmonary disease [13]. Currently, there are no clinical reports suggesting an association between TSP-1 and pulmonary hypertension (PH). Anatomic observations have shown that TSP-1 is up-regulated in the lung vasculature of mice exposed to chronic hypoxia [14] but its physiologic role in this setting is currently not known.

TSP-1^-/- ^mice developed pulmonary vascular smooth muscle cell hyperplasia spontaneously [15]. In addition, pulmonary artery endothelial cells undergoing cyclic stretch produce proteins that inhibit pulmonary artery smooth muscle cell growth and TSP-1 was responsible, at least in part, for this growth inhibition [16].

In this context, the purpose of the present manuscript was to describe the pulmonary vascular changes in TSP-1^-/- ^mice exposed to chronic hypoxia since chronic hypoxia induces pulmonary vascular remodeling and PH in rats and mice. [17-29]. Here we show that in the absence of the TSP-1 gene, mice were protected against chronic hypoxia-induced PH when compared to controls. TSP-1^-/- ^mice had lower right ventricular systolic pressures (RVSP), right ventricle/left ventricle + septum ratios (RV/LV+S), and less vascular remodeling than in WT controls. TSP-1^-/- ^mice also had decreased acute pulmonary vasoconstriction in response to U-46619 and acute hypoxia. Our data suggested that TSP-1 might play an active role in the pathogenesis of PH associated with hypoxic lung disease.

## Methods

### Animals

WT 129/SV male mice from Charles River Laboratories (Wilmington, MA) weighting 20-25 g were placed in a tightly sealed hypoxic chamber and provided with diet and water *ad libitum*. 129/SV TSP-1^-/- ^mice were a kind gift from Dr. Jack Lawler and were bred and genotyped as previously described [1]. TSP-1^-/- ^mice have been found to be susceptible to lung infection with *D Streptoccocus and Pasteurella pneumotropica *[1]. To help prevent this infection, a possible confounding factor in this research, the animals were treated with Sulfamethoxazole and Trimethoprim (200/40 mg - pediatric suspension-generic brand -50383082416). The only animals that received antibiotics were the TSP-1^-/- ^mice. They only received antibiotics while they were housed prior to hypoxia. Neither WT nor TSP-1^-/- ^mice group received antibiotics while they were exposed to hypoxia. A total of 93 animals were studied with a minimum of 4 per group.

### Hypoxic Chamber

The Subcommittee on Research Animal Care (SRAC) at Massachusetts General Hospital approved the protocol used in this study. The chronic hypoxia chamber is capable of housing 24 animals and has been described previously [30]. Oxygen concentrations were maintained at FiO_2 _0.21 or at normobaric hypoxia FiO_2 _0.1 +/- 0.05 by controlling the flow rates of compressed air and nitrogen. Gases were circulated through the chamber by a fan. FiO_2 _of 0.1 produces an arterial PO_2 _of less than 60 mm Hg [31]. Chamber oxygen concentration was tested daily by mass spectrometry [17]. CO_2 _absorbent (barium hydroxide lime, USP; Warren E. Collins, Braintree, MA) was added to keep the CO_2 _content <0.4%. Husbandry was done three times a week.

### Measurements of right ventricular pressure

Before pressure measurements, at least one hour of recovery was allowed following removal from hypoxia. Animals (Wild type and TSP-1^-/-^) were removed from hypoxia and anesthetized with Ketamine (90 mg/kg) and Xylazine (10 mg/kg) intraperitoneally. Animals were placed on a thermoregulated surgical plate and body temperature was monitored and maintained at 37°C. Right ventricular pressure was measured with the use of a single lumen catheter (0.012" × 0.016" silicone tubing; Specialty Manufacturing Inc., Saginaw, MI) through the right external jugular vein. Immediately after placement of the catheter into the right ventricle, RVSP was measured with a Gould pressure transducer positioned at the midthorax of the animal and a Gould multichannel recorder (Gould Inc., Cleveland, OH). Proper catheter location was verified by the waveform of the pressure tracing. The catheter positioning was confirmed by necropsy. After obtaining the right ventricular systolic pressure, animals were sacrificed and used immediately for the determination of right ventricular hypertrophy, hematocrit, and lung pathology. Animals were euthanized by exsanguination. This method is consistent with the Panel on Euthanasia of the American Veterinary Medical Association.

### Acute pulmonary vasoconstriction challenges

U-46619 (Cayman Chemical; Ann Arbor, MI) was diluted following manufacturer's instructions. Animals were anesthetized and catheterized through both external jugular veins following the protocol described above. We infused U-46619 at 0.1 ug/Kg [32] trough the left jugular vein and measured the acute changes in RVSP thought the right jugular vein. Percent increased in RVSP immediately after U-46619 was calculated as RVSP post U-46619 - baseline RVSP/RVSP post U-46619 × 100. To carry out the acute hypoxic challenge, animals were anesthetized and right-heart catheterized followed by exposure to FiO_2 _0.1 in a closed, custom-made Styrofoam chamber. Changes in RVSP were measured 5, 10, and 15 minutes after the beginning of the exposure to hypoxia. Acute FiO_2 _of 0.1 was achieved by using a commercial available mix (Airgas; Hingham, MA) and acute oxygen concentration was measured every 5 minutes using mass spectrometry as described above.

### Measurement of right ventricular hypertrophy

The degree of right ventricular hypertrophy was measured by the ratio of the weight of the right heart to left hear heart. After removal of atria, the hearts were dissected and then the right ventricle (RV) was separated from the left ventricle and septum (LV+S) under a dissecting microscope (Braintree Scientific, Braintree, MA). The dissected wet samples were weighed after drying at 90°C for 48 hr to obtain the ratio of RV/LV+S. We have previously shown that drying the hearts for additional days does not remove any additional water [17,30].

### Evaluation of the pulmonary vasculature

The degree of pulmonary remodeling was assessed by measuring the percent wall thickness of the vessels (%WT) indexed to terminal bronchioles and intra-acinous vessels (vessels indexed to respiratory bronchiole or alveolar ducts) and the ratio of thick-walled vessels to total number of intra-acinous vessels (% thick) as previously described [17]. Briefly, the left lung of each animal was perfusion-fixed with 10% natural formalin at a pressure of 90 cm H_2_O for the pulmonary artery and 30 cm H_2_O for the trachea and then preserved in fixative for at least 48 hours. Paraffin-embedded lung tissues were sectioned (4 μm thick) and stained with hematoxylin-eosin and elastin staining (Verhoeff Van Gieson) for light microscope examination. For the measurement of the wall thickness of the vessels, a computer imaging analysis software (IPLab; Scanlytics Inc., Fairfax, VA) was used to capture images of individual pulmonary arteries with digital camera (Spot; Diagnostic Instruments Inc., Sterling Heights, MI), mounted on a light microscope and linked to a computer. To determine the percent thick-walled vessel (%TW), external diameter (ED) and internal diameter (ID) (defined as the distance between two diametrically opposed external or internal elastic lamina) was measured. The medial thickness of the vessel wall is defined as distance between inner and outer elastic lamina. The average of two measurements corresponding to the largest and shortest ED and ID was determined. %WT was calculated as (ED-ID)/ED × 100. Percent thick-walled peripheral vessels (%TWPV) was expressed as the number of thick-walled intra-acinous vessels divided by the number of thick-plus thin-walled vessels × 100. Vessels were considered thick-walled if they contained an internal and external lamina for greater than 50% of the circumference of the vessel.

### Statistical Analysis

Statistical Analysis was performed using Statview 4.5 (Abacus Concepts, Inc and SAS Institute, Inc., Cary, NC). The pulmonary hemodynamic measurements, hematocrit measurements, percent wall thickness, and percent thick vessels after hypoxia were expressed as mean ± standard error. Analysis of variance (ANOVA) was used to assess the statistical significance of the differences followed by Fisher's PLDS test for the values identified by anova. We considered a p < 0.05 statistically significant.

## Results

### TSP-1^-/- ^mice had lower Right Ventricular Systolic Pressures (RVSP) and Right Ventricle/Left Ventricle + Septum (RV/LV+S) ratios than WT controls when exposed to 6 weeks of hypoxia

**Figure 1:**
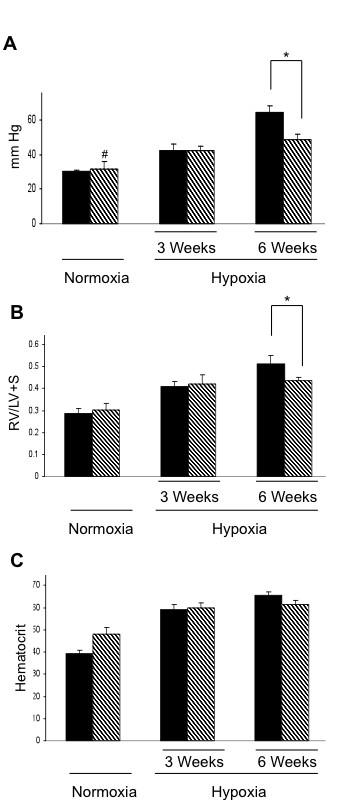
**TSP-1^-/- ^mice have less pulmonary hypertension in response to hypoxia**. **(A) **Right ventricle systolic pressures (RVSP). **(B) **Right ventricle/left ventricle + septum ratios (RV/LV+S). **(C) **Hematocrits. * = p < 0.05 TSP-1^+/+ ^black bar; TSP-1^-/- ^gray bar.

In normoxia, there was no difference in the RSVP between TSP-1^-/- ^mice and WT counterparts. However, we found that TSP-1^-/- ^mice failed to reach the same RVSP and RV/LV+S ratios as WT mice did after 6 weeks of hypoxia (Figures 1A and 1B). Hematocrits from WT and TSP-1^-/- ^mice confirmed that both groups were equally hypoxic throughout the experiment (Figure 1C) and that hematocrits were not affected by the absence of the TSP-1 gene.

### TSP-1^-/- ^mice developed less pulmonary vascular remodeling in response to chronic hypoxia than their WT controls

**Figure 2:**
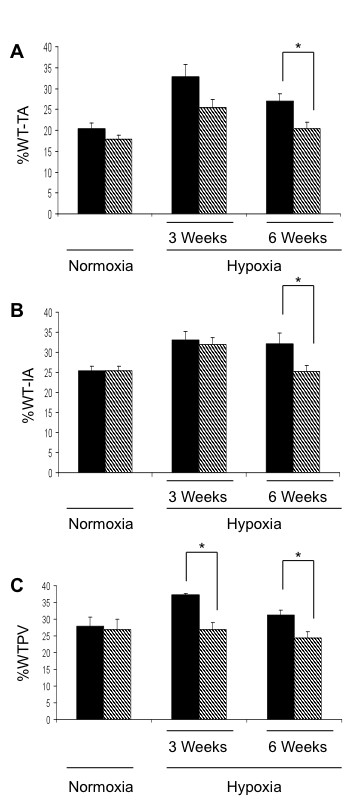
**TSP-1^-/- ^mice have less pulmonary vascular remodeling in response to hypoxia**. **(A) **Percent wall thickness of the vessels (%WT) indexed to terminal bronchioles vessels. **(B) **Percent wall thickness of the vessels (%WT) indexed to intra-acinous vessels. **(C) **Ratio of thick-walled peripheral vessels to total number of intra-acinous vessels (% TWPV). * = p < 0.05.

Pulmonary vascular remodeling, measured as %WT in terminal bronchial arterioles and intra-acinous vessels, was significantly less in TSP-1^-/- ^than in WT controls in response to 6 weeks of hypoxia (Figure 2A and 2B). Likewise, percent thick-walled peripheral vessels (%TWPV) remained unaffected in TSP-1^-/- ^mice (Figure 2C) exposed to hypoxia.

### TSP-1^-/- ^mice had less acute pulmonary vasoconstriction in response to challenges than WT mice

**Figure 3:**
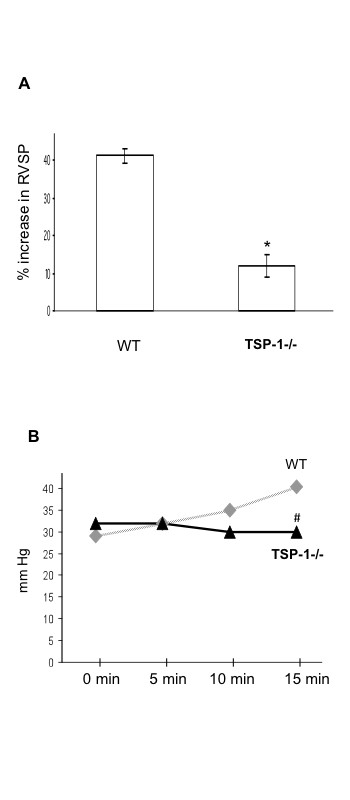
**TSP-1^-/- ^mice have less acute pulmonary vasoconstriction than WT controls**. **(A) **Immediate percent increase of RVSP in response to 0.1 ug/kg IV of U-46619. **(B) **Increase in RVSP after 5, 10, and 15 minutes of acute hypoxia in WT (gray line) and TSP-1^-/- ^mice (black line). * = p < 0.05 vs. WT. # p < 0.05 vs. WT at 15 minutes.

To further explore the TSP-1^-/- ^mice pulmonary vasculature decreased reaction to chronic hypoxia we investigated their response to acute pulmonary vasoconstrictors. We used U-46619 and FiO_2 _0.1 (Figures 3A and 3B). TSP-1^-/- ^mice showed a modest % increase in RVSP (11.9% ± 3.05 Vs 41.3% ± 1.99 p < 0.05) in response to U46619. TSP-1^-/- ^mice had significantly lower rise in RVSP after 15 minutes of acute hypoxia when compared to their WT controls (41.8 mmHg ± 2.3 Vs 31 mmHg ± 0.6; p < 0.05)

## Discussion

Although recent advances in the treatment of PH have prolonged the survival of patients suffering from this disease, the basis for new and better therapeutics, as well as a possible cure, are limited by our own capacity to comprehend the pathogenetic processes that control pulmonary vascular remodeling and PH. This implies the need for new observations, ideas, and fresh hypotheses that might lead to new molecular targets for the treatment of PH.

Thrombospondins are matricellular proteins composed of multiple well-defined structural repeats. TSP-1 is both antiangiogenic and proangiogenic. Through its central type 1 repeat, TSP-1 is a potent antiangiogenic factor. However, its N-terminal domain has pro-angiogenic activity. These functional differences have been explained on the ability of TSP-1 domains to interact with different extracellular receptors.

Recent reports have shown that TSP-1 is up-regulated by hypoxia in pulmonary arteries in piglets [33]. Likewise, TSP-1 has been found in intrapulmonary arteries in hypoxia-induced pulmonary hypertension in mice [14]. Nonetheless, the function of TSP-1 in the hypoxic pulmonary vasculature is presently unknown.

Crawford et al. reported that TSP-1^-/- ^mice had pulmonary vascular smooth muscle cell hyperplasia and that the abnormalities found in TSP-1^-/- ^mice regressed once the animals were treated with a TSP-1-derived peptide [15]. Our own previous observations were in line with these reports in that TSP-1 produced by the pulmonary endothelium inhibited pulmonary smooth muscle growth [16]. This suggested to us that the absence of TSP-1 could result in increased pulmonary vascular remodeling in animals exposed to chronic hypoxia. However, here we show that TSP-1^-/- ^mice were less responsive to the development of PH when exposed to chronic hypoxia. TSP-1^-/- ^mice failed to reach the same right ventricle systolic pressures (RVSP) and right ventricle/left ventricle +septum ratios (RV/LV+S) as compared to WT controls at 6 weeks of hypoxia (Figure 1). In addition, TSP-1^-/- ^mice developed less vascular remodeling than WT controls after chronic hypoxia (Figure 2).

We further explored the lack of pulmonary vascular remodeling and PH in TSP-1^-/- ^mice by challenging them with acute pulmonary vasoconstrictors (Figure 3). TSP-1^-/- ^mice had less acute pulmonary vasoconstriction than WT controls in response to both acute hypoxia (FiO_2 _0.1) and a thromboxane mimetic agent. This argued a possible role of TSP-1 in acute smooth muscle cell tone and contraction. The lack of acute hypoxic response may have contributed to the diminished pulmonary vascular remodeling changes with chronic hypoxia.

Lawler et al. reported the first TSP-1^-/- ^mouse [1]. They showed that the absence of the TSP-1 gene was associated with the development of pneumonia. In our study, we treated our animals with antibiotics prior to chronic hypoxia in order to prevent any pulmonary infections. Antibiotic treatment was stopped immediately before hypoxia to prevent any effects of the medication on the response to chronic hypoxia. Although, this did not prevent completely the development of pneumonia, it reduced the infection to a very small segmental area of the right lung. Though this minimal airspace disease may have contributed to a higher degree of hypoxemia, this did not result in a larger rise in hematocrit as compared to WT mice suggesting there was no significant difference in the degree of hypoxia (Figure 1). When we measured RVSP and vascular remodeling in normoxic TSP-1^-/- ^mice treated with antibiotics we were not able to confirm any pulmonary vascular remodeling compared to the pulmonary vascular smooth muscle cell hyperplasia reported in TSP-1^-/- ^mice previously by Crawford et al [15] (Figure 2). This suggests that the pulmonary smooth muscle cells hyperplasia found by Crawford et al. could have been a simple response to the lung inflammation caused by the lung infection. The pulmonary vascular remodeling seemed to have ceded when antibiotics prevented the pneumonia. The use of antibiotics might be confounding variable in our study. However, the antibiotic we chose to use has a half-life of 24 minutes in mice [34]. Second, the half-life and the length of our study design (6 weeks) allowed us to make the assumption that the animals cleared any residual antibiotic. Finally, there is currently no evidence linking Sulfamethoxazole or Trimethoprim with reduced smooth muscle cell contractility.

Even though we previously found that TSP-1 inhibited pulmonary smooth muscle cell growth *in vitro*, the present data shows that TSP-1 seems to stimulate pulmonary smooth muscle cell growth *in vivo*. Although TSP-1 suppresses wound healing and granulation tissue formation in the skin of transgenic mice [35]; antibody blockade to TSP-1 reduces neointima formation in balloon-injured rat carotid artery [36]. This suggests not only that TSP-1 functions are different *in vitro *and *in vivo*, but also that its functions *in vivo *depend upon the physiological system and cell type studied.

At this point we can only speculate on the role of TSP-1 in the pulmonary vasculature of chronic hypoxic mice. Recently, TSP-1 has been recognized as a powerful inhibitor of the Nitric Oxide (NO)- cyclic GMP (cGMP) signaling pathway in vascular cells [37,38]. The lack of response in TSP-1^-/- ^mice to hypoxia-induced pulmonary hypertension could be explained on the basis of an NO/cGMP "constitutively active" pathway due to the lack of TSP-1 antagonism. This points to a pro-hypertensive role for TSP-1 *in vivo*. Interestingly, Isenberg has proposed that the high levels of circulating TSP-1 associated with solid tumors could enhance tumor perfusion through TSP-1's hypertensive activity [39]. The same group reported that the systemic hypertensive response to epinephrine is attenuated in TSP-1^-/- ^mice [40]. Our observations suggest that TSP-1 function in sustaining vascular tone in the systemic circulation could be reproduced in a hypoxic model of pulmonary hypertension.

## Conclusion

TSP-1^-/- ^mice exposed to chronic hypoxia showed lower RVSP, and RV/LV+S ratios as well as less pulmonary vascular remodeling when compare to WT. These changes can be explained by the lack of response of TSP-1^-/- ^mice to acute pulmonary vasoconstrictors. We interpret these findings to mean that TSP-1 or TSP-1-dependent signaling modules play an active role in regulating the vascular reactivity of pulmonary vessels. TSP-1 or TSP-1-regulated signaling cascades could be exploited as new molecular targets in the treatment of pulmonary vascular diseases.

## Abbreviations

TSP-1: Thrombospondin-1; WT: Wild Type; FiO_2_: Fraction of Inspired Oxygen; RVSP: Right Ventricle Systolic Pressure; RV/LV+S: Right Ventricle/Left Ventricle + Septum; PH: Pulmonary Hypertension; SRAC: Subcommittee on Research Animal Care; PO_2_: Pressure of Oxygen; %WT: Percent Wall Thickness; ED: External Diameter; ID: Internal Diameter; %TWPV: Percent Thick-Walled Peripheral Vessels; ANOVA: Analysis of Variance; NO: Nitric Oxide; cGMP: cyclic 5' guanosine monophosphate.

## Competing interests

DQ now is employed by Novartis Pharmaceuticals. Novartis did not support this work and was not involved in the planning or execution of this work.

## Authors' contributions

CDO designed experiments, carried out genotyping, acute and chronic hypoxic studies, pulmonary morphometrics and statistical analyses, and drafted the manuscript. LY participated in the pulmonary morphometrics analyses. EA participated in the design of the study and helped to draft the manuscript. CAH participated in the design of the study, helped to draft the manuscript, and approved the final version of the manuscript to be published. DAQ conceived of the study, participated in the design of the experiments, participated in the statistical analyses; helped to draft and approved the final version the manuscript. All authors read and approved the final manuscript.
